# The Bed Feels Hard*A reprint of this article will be published, which those who are interested can have by sending three two-cent stamps.—Ed.

**Published:** 1897-12

**Authors:** H. C. Morrow

**Affiliations:** Austin, Texas


### "THE BED FEELS HARD."*

H. C. Morrow, M. D., Austin, Texas.

Recently I had a patient who complained that "the bed felt too hard," although the bed was as comfortable as it could be made. I knew from past investigations that Arnica, Baptisia, Pyrogen, and some others had this symptom, but the question was what others, and so I went to work to find out. Looking at Allen's *Bœnninghausen's Therapeutic Pocket Book*, I was astonished at the wealth of material there offered under the heading, "Hard Bed, Sensation of," page 162, but was surprised more to find that Baptisia and Rhus were not mentioned at all. The remedies there given under this rubric are: Acon., ARN., Bry., Caust., *Con.*, *Dros.*, Graph., *Kali-c.*, Mag-c., Mag-m., Nux-mos., Nux-vom., *Phos.*, Plat., Sabad., SIL., Stann., Sulph., Tarax., Thu., Verat-alb.

Bœnninghausen's original *Therapeutic Pocket Book* gives the same remedies with Magnes. and Megnetis-caust. added under the rubric, "Sensation as if one had been lying on a board," which is merely a variation of terms. Never having prescribed any of these remedies except Arnica for this condition, and not finding in my notes where any one else had any of them indicated for the symptom, I concluded to go to the fountain-head and search the *Materia Medica*. To my surprise I found the symptom or something similar under a majority of the respective remedies under this heading, but why several of them were included does not appear in the provings.

As some of the readers of The Homœopathic Physician may not have the time, and others lack the inclination to make this search for themselves, I will give the results of my work for their perusal and future reference. I will premise my observations by first stating that in order for a patient or

* A reprint of this article will be published, which those who are interested can have by sending three two-cent stamps.—Ed.

prover to have this symptom, he must also have the symptom, "bruised, sore feeling in some or all parts of the body."

Not all the remedies that have bruised, beaten or sore feeling have also the symptom, "the bed feels hard," but all the remedies which have this latter symptom have also the symptom, "he feels sore, beaten, or bruised in some or all portions of the body."

The king remedies, if I may be allowed the expression, which have this symptom are Arnica and Baptisia, with, perhaps, Pyrogen a close second. Although Bœnninghausen assigned the greatest numerical value to Silicea, yet the preponderance of verifications as reported in our journals is largely in favor of the former named remedies.

The symptoms of Arnica and Baptisia are as follow:

Everything upon which he lies seems too hard, ARNICA.

Weary, bruised, sore, great weakness, must lie down, yet the bed feels too hard, ARNICA.

Tired feeling, as after hard work, or as if beaten, ARNICA.

Indescribable pain in the foot, as from internal uneasiness, and as if the bed were too hard, which compels him to lay the foot here and there and to move it about, *Arnica*.

Each time after waking from the nightmare the parts on which he lay soon became exceedingly painful, Baptisia.

The parts on which he is lying feel sore and bruised, BAPTISIA.

After lying not more than ten minutes upon the back the sacral region became intolerably painful, as though he had lain on the barn floor all night, Baptisia.

Intolerance of pressure on all parts on which pressure is made; could not rest back against chair without pain from the pressure; obliged to change sitting position from the same cause; even the feet became equally painful from resting on the floor, Baptisia.

Feels as if lying on a board; changes position often, bed feels so hard, BAPT.

Silicea does not have the symptom, "the bed feels hard," but other symptoms of its pathogenesis make it entirely probable that Bœnninghausen's generalization is correct.

Bruised feeling over the whole body, as if he had lain in an uncomfortable position, SILICEA.

The whole side of the body on which he is lying is painful, SILICEA.

The whole body is painful, as if beaten, SILICEA. It does not require a very violent stretch of the imagination to believe that to a person in this condition, "the bed would feel hard." But if Bœnninghausen gives Silicea the place of greatest importance under this heading, why should he omit entirely Ruta-grav.?

In Allen we find: All parts of the body upon which he lies, even in bed, are painful, as if bruised, RUTA. If the bed is hard under Silicea why should it not be hard under Ruta, when neither has the exact wording of this symptom in its proving? Taking up Bœnninghausen's list of remedies we find the symptoms as follows to warrant him in so placing them:

Pain as from a contusion in shoulders and hip joints after sleeping, as if the bed had been too hard, Aconite.

All the limbs seem bruised and paralyzed, as if he had lain upon a hard bed, *Bryonia.*

He cannot lie in the bed in the morning; everything he lies on hurts him, Bryonia.

At night cannot get a quiet position; cannot lie still a moment, *Causticum.*

At night the side, hip, and thigh upon which he lay were sore, as if bruised, or as if pressed; he was frequently obliged to turn over, *Causticum.*

Bruised pain in the arms and legs; severe bruised pain in all the limbs; he cannot get rested in any position, *Conium.*

Aching in all the limbs upon which he lies, as if the bed were too hard, and he had not enough to lie upon, Drosera.

Soreness from lying in bed, Graphites.

Bruised pain in the limbs and scapula upon which he lies, Graphites.

Bruised pain in the back during rest; bruised pain in all the muscles of the body; flesh feels tender all over as if skin were crushed, Kali-carb.

Bed felt as hard as though she were lying upon stones, and she constantly tossed about, Magnesia-carb.

Pain over the whole spine, as if beaten to a jelly, while lying on the back, Magnesia-mur.

The whole body is painful, as if beaten to a jelly, Magnesia-mur.

Pain in small of back and both hips, as if sore and beaten, Magnesia-mur.

She complained very much about feeling very sore and full of pain, as one who had been beaten, Nux-moschata.

If he lies upon a moderately hard substance for only a short time he immediately experiences pains in the parts upon which he lies, Nux-moschata. Parts upon which the patient lies ache as if sore, Nux-moschata.

Pain in all the limbs, as if beaten and bruised all over, *Nux-vomica.*

The upper and lower extremities are sore, as if she had lain on a hard bed, Nux-vomica.

The desire to sleep was great, but I could not find a comfortable position, Phosphorus.

Dull pain in the haunch bones in the evening, as if he had been lying upon too hard a couch; he had to alter his position all the time, Phosphorus.

The parts affected by cramp-like pain are painful to pressure, PLATINUM.

Bruised pain in arms, back, and thighs, as if beaten, Platinum.

Pains in back and small of back, as if bruised or broken, Platinum.

Intense but transient bruised pain in various parts of the body, Sabadilla.

In the morning when waking more weary than ever; all her body feels painful, as if she had been resting on blocks of wood, Sabadilla.

Bruised pain in all the limbs; pain as if beaten in the back and lower extremities, Stannum.

Heaviness in the back in the morning, as if he had been lying uncomfortably, Sulphur.

Pain in the back and small of the back, as if beaten to pieces, *Sulphur*.

Bruised sensation in the small of the back, on account of which he could not sleep, SULPHUR.

Great weariness of the limbs, so that he could find no comfortable position in bed, Sulphur.

In the morning on awaking bruised feeling of the whole body, Sulphur.

All the limbs are painful when touched, or when in an uncomfortable position, Taraxacum. During rest intolerable tearing pains in lower extremities, *Taraxacum*.

Parts lain on painful, THUJA.

Constant uneasiness in sleep, because he always wishes to lie in another place, as the parts upon which he lies speedily become very painful, *Thuja*.

Pain consisting of a pressure or a bruised sensation in the muscular parts of the body, *Veratrum-album*. This is the evidence taken from the *Materia Medica*, after diligent search which best sustains Bœnninghausen's arrangement under this rubric.

Each one can judge of the value of the evidence for himself. In favor of the correctness of his arrangement the evidence justifies it undoubtedly with the larger number, but in regard to some of them we fail to see why they should be so designated.

There is not a single symptom in the proving of Verat-alb. for instance to show why it should be placed under the heading, "sensation as if lying on a board," or as if "the bed were too hard," and there is but little for Taraxacum, Stannum, Platinum, Conium, both of the latter of which are honored with the second rank.

One wonders when reading the proving of some of the other remedies why they were not included under the same heading, for example, Ruta-grav., already spoken of, and the following:

The parts of the body upon which he sits feel sore and bruised, Agaricus.

Back and limbs feel as if bruised while lying still in bed, *Pulsatilla.*

Tired aching, as from lying long in one position, *Ferr-met.*

Constant pains along the back, worse in those parts she had to lie on, *Ferrum-met.*

He awoke with a bruised pain over whole body, Spongia. Quite a number of remedies have this symptom which are not included above, to wit:

Bed feels so hard she cannot lie upon it, OPIUM.

At 5 A. M. mind active and cannot sleep again; bed feels so hard, Fagopyrum.

Tossing here and there, the bed seemed harder than usual, and I could find no place to rest, Euphrasia.

Everything upon which he sits or lies seems too hard, Lycopodium, Petroleum.

Pain in the sacrum, as if he had been lying upon too hard a couch, Mercurius.

Aching all over; bed feels hard; can lie but a few moments in one position; aching with soreness of the flesh, PYROGEN.

Awoke frequently with pain in the loins and hips, causing frequent change of position in bed, which seemed too hard, Pulsatilla-nuttallina.

Her bed feels too hard, Ferrum-phos. (Clinical symptom, Dr. Deschere, N. A. J. H., Feb., 1897.)

Gentry's *Concordance Repertory* gives Arsenicum in brackets as having this symptom, but if it has it, it must be a clinical symptom, because it is not in the proving of any of the *Materia Medicas*. Many consider this symptom as a very prominent one under Rhus-tox., but if so it is to a large extent clinical.

When lying upon side hip hurts, and when lying upon back small of back hurts, *Rhus-tox*.

Pain in small of back, as if bruised, compelling him to change his position constantly, Rhus-tox.

Pain as if bruised in the small of the back, as if bruised whenever he lies quietly upon it, or sits still, RHUS-TOX.

Among the newer remedies the following have more claims to be placed under this rubric than several which Bœnninghausen has placed there. Thus we have:

Soreness of all the muscles; general bruised feeling as if sore, ACTEA-RACEMOSA.

Must frequently change his position; body feels so sore, Badiaga.

Bruised feeling, as if broken all over body, EUPATORIUM-PERFOL.

Intense aching and soreness in thighs, HAMAMELIS. Bruised feeling in muscles of upper and lower extremities. HAMAMELIS. Bruised soreness over body, HAMAMELIS.

Feeling of soreness, as if beaten or pounded with prostration, Phytolacca.

Some of the remedies have symptoms which are directly opposite to those enumerated, which will be of interest in this connection.

Pain in small of back, worse when sitting still or lying, better when lying upon something hard, RHUS-TOX.

When stooping sudden pain in the back, as if struck by a hammer, relieved by pressing back against something hard, SEPIA.

Pain as if bruised in small of back, ameliorated by lying on something hard, NATRUM-MURIATICUM.

Simple pain in the whole of the back, as from weariness; it comes on while walking; movement avails nothing, but is relieved when he sits down, makes his back hollow and leans firmly against anything, Sabadilla.

I think Belladonna has the clinical symptom, "pain in the back, relieved by pressing against something hard," but am not certain. It is not in the proving.

Tired aching feeling in all the muscles of the body, relieved by lying on something hard, Sanicula (clinical symptom).

A peculiar symptom is: Pain as if bruised in those limbs and joints upon which he does not lie, *Rhus-tox*.

When lying head feels as if lying upon something hard, Mancinella.

The pillow feels as hard as iron, ARNICA, *Baptisia*, Phosphorus.

With all our multiplicity of repertories not one-half of the above symptoms can be found within the pages of all combined.

Let us hope that some day we will have a repertory that will contain *all* the symptoms of the materia medica under such headings that one can find any sensation, aggravation, or amelioration in any of our *Materia Medicas*.

---

### POSTSCRIPT.

(A subsequent letter to the Editor.)

I think I omitted one remedy that has the "bed too hard" symptom, "sleepless, restless on account of bed feeling too hard, Tilia-europ." Also a symptom of Graphites, "soreness from lying in bed."

Please insert also the following where it will read the best:

"To sum up, I would assign under this heading to the first rank, Arnica, Baptisia, and probably Pyrogen. Regarding the latter remedy clinical reports are not yet sufficient to justify its exact rank.

"In the second rank, Opium, Rhus, Ruta, Silicea, and Thuja.

"In the third rank, Bryonia, Causticum, Nux-vomica, Phosphorus.

"In the fourth rank, Acon., Dros., Euphra., Fagop., Ferro-ph., Graph., Lycop., Mag-c., Merc., Nux-mos., Petrol., Puls-nut., Sabad., Tilia-europ.

"Doubtful, Arsen., Mag-m., Plat., Sulph.

"Omit entirely from this rubric, Conium, Kali-carb., Stannum, Taraxacum, and Veratrum-alb."

[The foregoing admirable article is published with great pleasure, as it contains indications for remedies which are of the highest value to homœopathists. Dr. Morrow wonders at the absence of several remedies from Bœnninghausen's *Therapeutic Pocket Book*. He evidently overlooks the fact that these remedies were added to the *Materia Medica* after Bœnninghausen had published his *Pocket Book*. Dr. Morrow desires the Editor to give his own notes. This is hard to do. Dr. Morrow has covered the ground so thoroughly that there is little to add. The only notes we find are: Bed feels too hard in fever, Phos. Must rise from bed on account of throbbing in the head, Arg-n. Limbs feel bruised and paralyzed, as if from lying on hard bed, Bryonia. The remaining notes are either repetitions of Dr. Morrow's indications or else refer to other conditions not specially germane to the subject.—Ed.]

